# Host-Informed MaxEnt Modeling Reveals Distinct Global Distribution and Redistribution Patterns of Four Major *Fusarium graminearum* Species-Genotype Groups Under Current and Future Climates

**DOI:** 10.3390/foods15173096

**Published:** 2026-08-31

**Authors:** Hao Li, Baizhu Chen, Chaoyi Zhang, Junhua Yang, Jianhua Wang

**Affiliations:** 1 Institute for Agro-Food Standards and Testing Technology, Shanghai Academy of Agricultural Sciences, 1000 Jinqi Road, Shanghai 201403, China; m240100147@st.shou.edu.cn (H.L.); m240100146@st.shou.edu.cn (B.C.); m250100142@stu.shou.edu.cn (C.Z.); yangjunhua303@126.com (J.Y.); 2 College of Fisheries and Life Science, Shanghai Ocean University, Shanghai 201306, China

**Keywords:** *Fusarium graminearum* species complex, species-genotype groups, global distribution, climate change, future redistribution

## Abstract

The *Fusarium graminearum* species complex (FGSC) is a major causal agent of Fusarium head blight (FHB) and trichothecene contamination in cereal cropping systems worldwide. However, its major species-genotype groups exhibit distinct differences in environmental adaptability and spatial distribution. Using a host-informed MaxEnt modeling framework, this study compared the global potential environmental suitability of four major FGSC groups, namely *Fusarium asiaticum*-DON (Fa-DON), *Fusarium asiaticum*-NIV (Fa-NIV), *Fusarium graminearum*-DON (Fg-DON), and *Fusarium graminearum*-NIV (Fg-NIV), under the 1970–2000 baseline and four SSPs across four future periods. By integrating georeferenced occurrence records with continuous host-related, climatic, soil, topographic, and wind predictors, and applying four-fold spatial cross-validation, we identified distinct intergroup suitability patterns. Under current conditions, Fg-DON showed the broadest highly suitable area, whereas Fa-DON was the most restricted. Both *F. asiaticum* groups were centered in Asia, while Fg-NIV was concentrated mainly in Europe. Future projections indicated consistent expansion for Fg-DON and general contraction for Fa-DON. Fa-NIV remained comparatively stable, whereas Fg-NIV showed scenario-dependent contraction or expansion. These changes reflected regional redistribution rather than a uniform shift across groups. Collectively, our findings indicate that FGSC suitability should be interpreted as a set of differentiated, group-specific biogeographic patterns, which provide a more targeted scientific basis for surveillance strategies and regional risk assessment of FHB and trichothecene contamination under changing environmental conditions.

## 1. Introduction

The *Fusarium graminearum* species complex (FGSC) comprises a cluster of closely related fungal pathogens and is the primary causal agent of Fusarium head blight (FHB) in wheat, barley and other major cereal crops worldwide [1,2]. FHB epidemics lead to substantial grain yield loss and quality degradation, accompanied by severe contamination of type B trichothecenes including deoxynivalenol (DON), nivalenol (NIV), and their derivatives [3,4,5]. Mycotoxin pollution further triggers market restrictions and huge economic losses in global cereal production [6,7]. In recent decades, FGSC-associated diseases have caused increasingly severe production and economic damage across major cereal-producing regions, including China [8,9]. Therefore, spatially explicit comparisons of the potential environmental suitability of major FGSC groups are important for informing regional FHB surveillance and mycotoxin monitoring.

Accordingly, suitability modeled at the complex level may conceal group-specific environmental associations and spatial patterns [10,11,12,13]. Pathogenicity and trichothecene genotype differences further deepen the niche differentiation of FGSC subgroups [14,15]. Recent global syntheses also confirm that FGSC species composition, genotype structure and population diversity vary markedly across geographical regions and cropping systems [4,16,17,18]. These evidences indicate that FGSC consists of multiple ecologically distinct subgroups, rather than a single homogeneous entity. Accordingly, the overall suitability range of FGSC is actually a combination of multiple group-specific biogeographic patterns [19].

Such intrinsic heterogeneity presents a challenge for species distribution modeling (SDM). Global occurrence databases and regional surveillance studies have improved the characterization of FGSC biogeography, but direct global comparisons among species–trichothecene genotype groups remain limited [20,21]. Pooling occurrence records at the complex level can identify broad areas of environmental suitability while obscuring differences in the environmental associations and spatial patterns of individual groups. This limits the value of pooled models for determining which groups should be prioritized in regional sampling and toxin-genotype surveillance. The central question is therefore whether the major FGSC species-trichothecene genotype groups exhibit distinct current and future patterns of potential environmental suitability.

Maximum Entropy (MaxEnt) is a widely used presence-background SDM method that estimates relative environmental suitability from occurrence records and environmental predictors [22,23,24]. For FGSC and FHB risk modeling, previous studies mainly adopted climate-driven frameworks and confirmed that temperature and precipitation are key drivers of pathogen distribution [25]. Climate change can alter pathogen suitable habitats, infection windows and mycotoxin risks in cereal agroecosystems [26,27]. Existing climate-based SDMs have improved our understanding of climatic constraints on FGSC suitability [28], but host availability and crop composition have received less explicit attention.

A major limitation of climate-only SDMs for crop pathogens is their limited representation of host availability and infection context. FHB infection depends on the coincidence of viable inoculum, susceptible cereal tissues—particularly around anthesis—and favorable warm, humid, or rainy conditions [3,5,6,26]. Crop residues and cropping history further influence inoculum persistence and availability [29]. At broader spatial scales, the distribution and composition of cereal crops can therefore provide information on host-resource availability. Empirical studies have shown that wheat-, rice-, and maize-based cropping systems are associated with distinct FGSC population structures and trichothecene genotype patterns [30,31], while gramineous weeds may serve as alternative hosts [32]. However, gridded climate and crop layers characterize broad environmental and host-resource contexts rather than field-scale processes such as crop phenology, residue management, cultivar resistance, and fungicide use.

Herein, “host-informed” refers to the inclusion of two continuous indicators derived from SPAM2020 physical-area layers: the total area of wheat, barley, maize, and rice (host_total_log1p) and the relative composition of wheat-barley and rice cultivation (wet_dry_share). These variables were incorporated as predictors alongside climatic, soil, topographic, and wind variables; they were not used as a binary host mask or as evidence that FGSC was absent from grids without occurrence records. Thus, the framework evaluates how broad host-resource gradients are associated with modeled suitability while retaining the presence-background interpretation of MaxEnt. The outputs represent potential environmental suitability rather than realized occupancy, disease severity, or mycotoxin contamination.

In this study, we used calibrated group-specific MaxEnt models to compare the global potential environmental suitability of four major FGSC species-trichothecene genotype groups: *Fusarium asiaticum*-DON (Fa-DON), *F. asiaticum*-NIV (Fa-NIV), *Fusarium graminearum*-DON (Fg-DON), and *F. graminearum*-NIV (Fg-NIV). Occurrence data and continuous host, climatic, soil, topographic, and wind predictors were integrated to: (1) characterize differences in modeled suitability among the four groups under the 1970–2000 baseline; (2) identify group-specific predictors associated with these suitability patterns; and (3) quantify projected changes in continuous suitability, highly suitable area, and the expansion, contraction, and centroid shifts of modeled suitability under BCC-CSM2-MR projections for SSP126, SSP245, SSP370, and SSP585 across 2021–2040, 2041–2060, 2061–2080, and 2081–2100. The resulting comparisons are intended to provide a spatial reference for prioritizing future group-specific surveillance under climate change.

## 2. Materials and Methods

### 2.1. Study Data and Preprocessing

This study defined four species-genotype groups as *Fusarium graminearum*-DON (Fg-DON), *Fusarium graminearum*-NIV (Fg-NIV), *Fusarium asiaticum*-DON (Fa-DON), and *Fusarium asiaticum*-NIV (Fa-NIV), and constructed host layers using four cereal crops: wheat, barley, maize, and rice. Together, these four species-genotype groups included 15,239 records, representing 91.4% of all records in the FGSC database (https://edelponte.shinyapps.io/FGSCdb/, accessed on 18 August 2026). Within the combined *F. graminearum* 90.2% of the subset total [20]. Focusing on these dominant groups and primary hosts allowed us to capture the core species-host structure in the database while minimizing noise from rare taxa, non-primary hosts, and sparsely annotated genotypes. Detailed occurrence-processing counts are provided in Supplementary Table S1, and the retained occupied cells are shown in Figure S1.

Records with invalid coordinates were removed. The remaining records were assigned to the 0.5° modeling grid, and each occupied cell was represented by one presence, leaving 117, 133, 803, and 42 occupied cells for Fa-DON, Fa-NIV, Fg-DON, and Fg-NIV, respectively. Original record counts within cells were retained only for auditing and were not used as predictors or sample weights.

Three categories of datasets were used in this study: (1) georeferenced occurrence records with clear species and genotype information; (2) multi-dimensional environmental predictors including climate, soil, topography and wind; (3) future bioclimatic and wind data were obtained for BCC-CSM2-MR under SSP126, SSP245, SSP370, and SSP585 for 2021–2040, 2041–2060, 2061–2080, and 2081–2100 due to its excellent performance in simulating temperature and precipitation over East Asia [33,34,35,36]. MaxEnt calibration was conducted across a common terrestrial background defined by the spatial overlap of all predictor layers, thereby maintaining a consistent environmental domain for presence–background comparisons [23,37]. Detailed data sources are summarized in Table 1, and full variable descriptions are provided in Supplementary Table S2.

The predictor set covered multiple ecological dimensions. Climatic variables describe the temperature and moisture conditions that constrain pathogen survival, infection opportunities, and mycotoxin-related risk. Host-related variables reflect the spatial availability of cereal resources and the relative dominance of different species-genotype groups across regions, thereby influencing their establishment opportunities and distribution patterns. Topographic and soil variables were included because terrain and edaphic conditions can modify local hydrothermal regimes and near-ground microenvironments, thereby affecting both host performance and pathogen persistence. Wind-related variables were further considered as coarse proxies for atmospheric connectivity and dispersal potential. Together, these predictor groups were used to characterize the joint climatic, host-related, and environmental constraints on FGSC suitability. For future projections, time-varying bioclimatic and wind variables were replaced with BCC-CSM2-MR SSPs data, while host, soil, elevation, and slope layers were held constant.

Model calibration was conducted over a common terrestrial background defined by the spatial intersection of the predictor layers. Host availability was incorporated explicitly as continuous environmental covariates rather than being used to delimit the geographic calibration domain. Crop information was obtained from SPAM2020 v2.2, which provides gridded estimates of the physical area of major crops worldwide [38]. Physical-area layers for wheat, barley, maize, and rice were harmonized to the common model grid and used to derive two host-related predictors.

Host availability was further represented by two derived variables. The first was total host resource intensity,

(1) $$host\_total\_log1p=\ln\left(1+A_{wheat}+A_{barley}+A_{maize}+A_{rice}\right)$$

The second variable represented the relative ratio of dryland farming to wetland farming within a given region,

(2) $$wet\_dry\_share=\{\begin{array}{c} \frac{A_{wheat}+A_{barley}}{A_{wheat}+A_{barley}+A_{rice}},A_{wheat}+A_{barley}+A_{rice}>0,\\ \;\;\;\;\;\;\;\;\;\;\;\;\;\;\;\;\;\;\;\;\;0.5{\;\;\;\;\;\;\;\;\;\;\;\;\;\;\;\;\;\;,A}_{wheat}+A_{barley}+A_{rice}=0. \end{array}$$

where $A_{wh\mathrm{eat}},\;A_{\mathrm{barley}},{\;A}_{\mathrm{maize}},\;A_{\mathrm{rice}}$ denote the physical areas of wheat, barley, maize, and rice, respectively, in each grid cell. When wheat, barley, and rice were all absent, wet_dry_share was assigned a neutral value of 0.5, including maize-only and no-four-crop cells. In no-four-crop cells, host_total_log1p was equal to 0 and therefore retained the information that none of the four focal hosts was present. A wet_dry_share value of 0 represented cells containing rice but no wheat or barley. These two host-related variables were then incorporated together with climatic, edaphic, topographic, and wind-related predictors into subsequent variable screening and model calibration.

### 2.2. MaxEnt Model Calibration and Spatial Validation

We adopted the MaxEnt algorithm for habitat suitability modeling, which is robust for presence-only datasets. Potential environmental suitability was modeled using MaxEnt version 3.4.4 with complementary log-log (cloglog) output [22,23,24,37]. Prior to model calibration, collinearity among the 46 candidate predictors was examined using pairwise Pearson correlation coefficients and variance inflation factors (VIFs). Predictors were screened using an absolute correlation threshold of |r| = 0.8, followed by iterative VIF analysis until all retained variables had VIF ≤ 5 [39]. This procedure yielded a common pool of 25 candidate predictors for the four FGSC groups.

Model calibration and evaluation were conducted using four-fold spatial cross-validation [40,41]. Predictor selection and parameter optimization were performed independently within each training partition before evaluation on the corresponding withheld spatial fold [40,42]. The study domain was divided into 7.5° × 7.5° checkerboard blocks and assigned to four spatial folds. In each iteration, one fold was retained for model evaluation and the remaining three folds were used for model calibration. Within each training partition, an initial MaxEnt model was fitted using the 25-variable candidate pool, and predictors were ranked according to permutation importance. Three candidate predictor subsets were then constructed by retaining the minimum number of variables required to reach cumulative permutation-importance thresholds of 80%, 85%, and 90%, hereafter referred to as P80, P85, and P90, respectively. Each subset contained at least three predictors. Predictor ranking and construction of the candidate subsets were repeated independently for each training partition before the corresponding withheld fold was used for evaluation.

The P80, P85, and P90 predictor subsets were evaluated in combination with alternative feature-class (FC) and regularization-multiplier (RM) settings. For Fa-DON, Fa-NIV, and Fg-DON, the candidate FC combinations were L, LQ, LQH, LQHP, and LQHPT, where L, Q, H, P, and T denote linear, quadratic, hinge, product, and threshold features, respectively. For Fg-NIV, L, LQ, and LQH were evaluated. Regularization multipliers were set to 0.5, 1.0, 1.5, 2.0, 2.5, 3.0, and 4.0. Candidate configurations were compared using the corrected Akaike information criterion (AICc) [43,44]. Models that did not converge within 1000 iterations or returned a non-finite AICc were excluded, and the configuration with the lowest AICc was retained within each training partition. The selected configuration was subsequently evaluated on the corresponding withheld spatial fold. Because model selection was repeated independently for each training partition, the selected predictor subset, FC, and RM were allowed to differ among folds. Model performance was summarized using training and test area under the receiver operating characteristic curve (AUC), the standard deviation of test AUC among folds, the minimum fold-specific test AUC, and the difference between training and test AUC [45].

After completion of the four-fold spatial evaluation, final models were fitted separately for each FGSC group using all available occupied grid cells. Predictor-subset construction and FC–RM selection followed the same criteria used during spatial cross-validation, and the converged configuration with the lowest AICc was retained as the final model for subsequent spatial projections. The selected final model configurations and predictor sets are summarized in Table 2. Predictor effects were characterized using percentage contribution, permutation importance, jackknife analysis, and response curves. Each final model configuration was additionally refitted using 100 bootstrap samples of the occurrence data. Bootstrap response curves were summarized using the mean response and empirical 2.5th and 97.5th percentiles. Bootstrap models were used for response-curve uncertainty analysis, whereas current and future suitability surfaces were generated from the final model fitted to the complete occurrence dataset.

### 2.3. Current and Future Suitability Projections and Resolution Sensitivity Analysis

Current environmental suitability was estimated using the 1970–2000 baseline conditions. Future climatic conditions were represented by the BCC-CSM2-MR global climate model under four CMIP6 Shared Socioeconomic Pathways (SSP126, SSP245, SSP370, and SSP585) and four future periods: 2021–2040, 2041–2060, 2061–2080, and 2081–2100 [34,35,36]. These combinations provided a common set of future climate conditions for evaluating changes in the modeled suitability of the four FGSC groups. The future analysis focused on changes associated with climatic and atmospheric conditions. Accordingly, bioclimatic and wind-related predictors were updated for each scenario and period, whereas soil properties, elevation, slope, and host-related predictors were retained at their baseline conditions. Host availability therefore corresponded to the crop distributions represented by SPAM2020 v2.2 throughout the future projections. The calibrated MaxEnt models were projected under each future climate combination, with clamping applied during model transfer [37].

Continuous cloglog predictions were used to represent environmental suitability. For comparisons of highly suitable areas, cells with cloglog values ≥ 0.8 were classified as highly suitable using the same criterion for all FGSC groups, scenarios, and periods. This threshold was used for categorical spatial comparison and was not interpreted as the probability of pathogen occurrence. Because the physical area represented by geographic grid cells varies with latitude, the area of each cell was calculated from its latitude-specific spherical surface area using a mean Earth radius of 6371.0088 km. Current and future high-suitability maps were compared to quantify stable, expansion, and contraction areas, and proportional changes in total high-suitability area were calculated relative to the corresponding current baseline.

Area-weighted geographic centroids were calculated from cells with cloglog ≥ 0.8 for each FGSC group and continent. For cell *i* with area *Aᵢ*, latitude *φᵢ*, and longitude *λᵢ*, the spherical coordinates were calculated as

(3) X=ΣAᵢcosφᵢcosλᵢ/ΣAᵢ,Y=ΣAᵢcosφᵢsinλᵢ/ΣAᵢ, Z=ΣAᵢsinφᵢ/ΣAᵢ

Centroid longitude and latitude were obtained as

(4) $$\lambda_{c}=atan2(Y,X)\;\phi_{c}=atan2\left(Z,\sqrt{X^{2}+Y^{2}}\right)$$

Current-to-future centroid-shift distances were calculated as great-circle distances using a mean Earth radius of 6371.0088 km.

The primary models were developed at 0.5° spatial resolution. Resolution sensitivity was assessed using additional models at 0.25° and 1° resolution. For resolution-sensitivity analyses, the predictor sets, feature classes, and regularization multipliers selected in the 0.5° main models were retained, while occurrence grids and environmental layers were reconstructed at alternative resolutions. Model discrimination among resolutions was compared using spatial test AUC, the difference between training and test AUC, and the minimum fold-specific test AUC. Similarity in continuous suitability patterns relative to the 0.5° predictions was quantified using Spearman’s rank correlation and Schoener’s D [46], whereas overlap among highly suitable areas was assessed using the Jaccard index. High-suitability area estimates and the direction of projected future change were also compared among resolutions.

**Figure 1:**
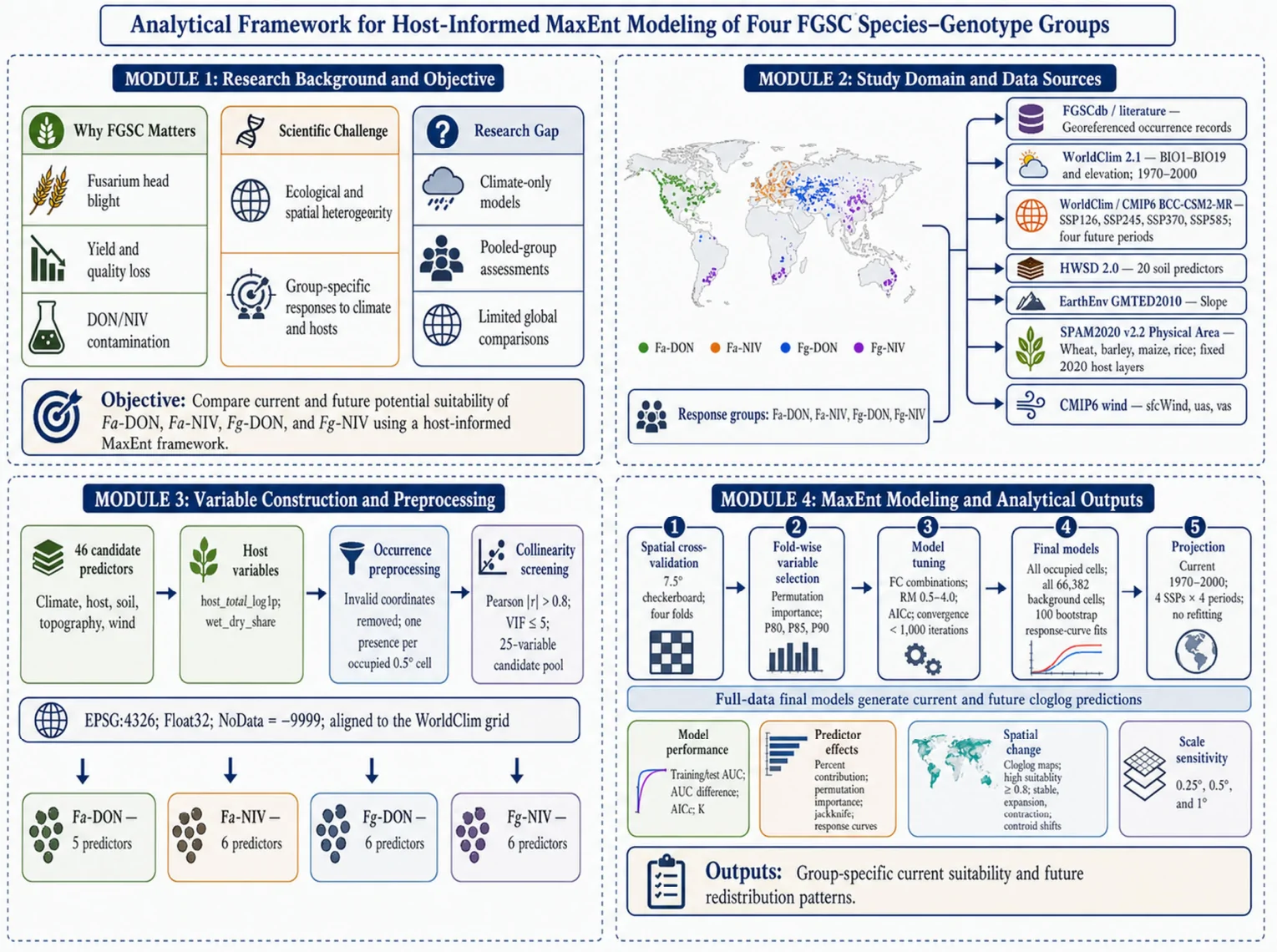
Technical workflow of host-informed MaxEnt modeling for four FGSC species-genotype groups.

The overall analytical framework is summarized in Figure 1. Georeferenced occurrence records of the four FGSC groups were integrated with climatic, soil, topographic, host-related, and wind predictors at the global scale. Occurrence data were aggregated to the 0.5° modeling grid, and candidate predictors were screened for collinearity before MaxEnt calibration. Model calibration incorporated spatial cross-validation, group-specific predictor selection, and optimization of feature classes and regularization multipliers, followed by fitting of the final models using the complete occurrence datasets. The calibrated models were subsequently used to characterize current environmental suitability and to project future suitability under four SSP scenarios and four future periods. Resolution-sensitivity analyses at 0.25° and 1° were additionally conducted to compare model performance and spatial predictions across scales. The complete modeling framework is presented in Figure 1.

## 3. Results

### 3.1. Predictor Screening, Model Calibration and Spatial Validation

Prior to model calibration, 46 candidate predictors covering climatic, soil, topographic, wind-related, and host-related environmental dimensions were evaluated for multicollinearity. After Pearson correlation filtering and iterative VIF analysis, 25 predictors were retained as the common candidate pool for subsequent MaxEnt calibration. These retained predictors included six climatic variables, multiple soil and topographic variables, three wind-related variables, and two host-related variables representing overall host resource availability and cropping-system composition (Table S2).

Four-fold spatial cross-validation showed consistently high predictive performance across the four FGSC species-genotype groups (Table 2; Table S3). The mean test AUC values were 0.9577, 0.9455, 0.9288, and 0.9625 for Fa-DON, Fa-NIV, Fg-DON, and Fg-NIV, respectively. The standard deviations of test AUC among spatial folds ranged from 0.0129 to 0.0270, and the minimum fold-specific test AUC values remained above 0.90 for all groups (0.9096–0.9385). The mean differences between training and test AUC ranged from 0.0120 to 0.0310, indicating that model performance remained stable when evaluated using spatially withheld occurrence data. Detailed fold-specific results, including training and testing sample sizes, selected configurations, and fold-level AUC values, are provided in Table S3.

The final full-data models differed among groups in predictor-set size, feature class, and regularization multiplier (Table 2). The selected Fa-DON model retained five predictors from the P90 set and used LQ features with RM = 0.5. The Fa-NIV model retained six predictors from the P90 set and used LQHP features with RM = 2.5. The Fg-DON model retained six predictors from the P90 set and used LQHPT features with RM = 2.0. The Fg-NIV model retained six predictors from the P85-derived set and used linear features with RM = 0.5. The corresponding numbers of effective parameters (K) were 10, 22, 96, and 6, respectively. The receiver operating characteristic curves of the final full-data models are presented in Figure S2, with training AUC values of 0.9822, 0.9704, 0.9515, and 0.9738 for Fa-DON, Fa-NIV, Fg-DON, and Fg-NIV, respectively.

### 3.2. Current Spatial Patterns of Highly Suitable Areas

**Figure 2:**
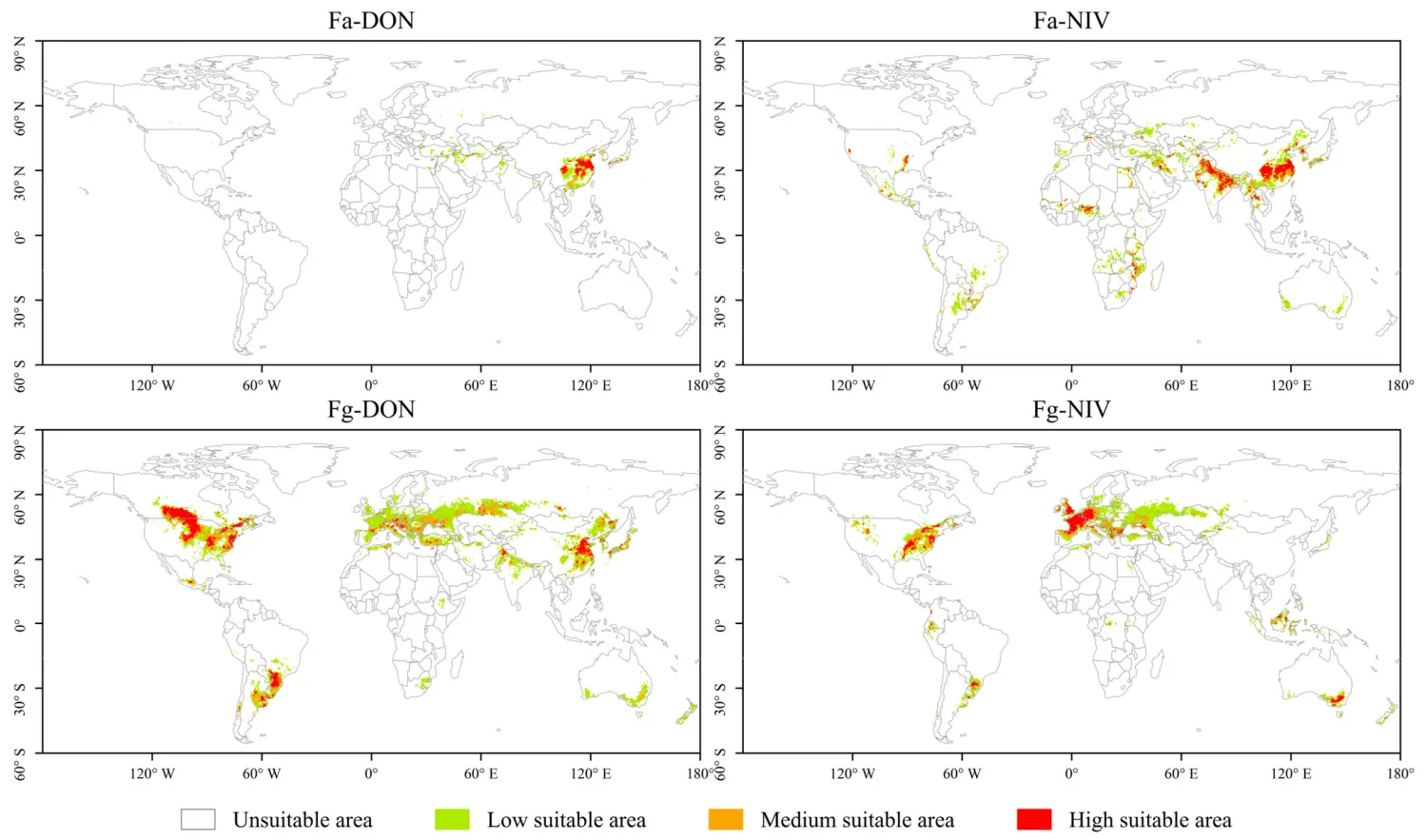
Current suitable areas of four FGSC species-genotype groups.

To facilitate spatial comparison among the four FGSC species-genotype groups, grid cells with cloglog values ≥0.8 were classified as highly suitable areas. This threshold was used as a consistent criterion to delineate areas with relatively high environmental suitability across groups, rather than as a direct probability of pathogen occurrence. Under current environmental conditions, the four FGSC species-genotype groups exhibited clearly differentiated global suitability patterns, despite sharing closely related evolutionary backgrounds and overlapping cereal hosts (Figure 2). The predicted suitability landscapes showed substantial variation in both geographic extent and spatial organization, indicating that the four groups occupy distinct environmental niches rather than representing a single homogeneous ecological entity.

Among the four groups, Fg-DON displayed the broadest global suitability pattern. Highly suitable areas were distributed across multiple major cereal-producing regions, showing a relatively extensive spatial footprint compared with the other groups. The distribution pattern of Fg-DON extended across several temperate agricultural regions, indicating a comparatively wide environmental tolerance under current conditions. In contrast, Fg-NIV exhibited a more concentrated suitability pattern, with highly suitable areas forming a narrower geographic distribution. Although both groups belong to *F. graminearum*, their contrasting suitability landscapes suggest substantial ecological differentiation between DON- and NIV-producing groups.

The two *F. asiaticum* groups showed a distinct regional pattern compared with the *F. graminearum* groups. Both Fa-DON and Fa-NIV were primarily associated with Asian cereal-growing regions, but their spatial extents differed markedly. Fa-DON showed the most limited suitability range among the four groups, whereas Fa-NIV occupied a considerably broader suitable area, suggesting stronger geographic expansion potential under current environmental conditions. This contrast indicates that trichothecene genotype differences within the same species background are associated with distinct spatial suitability structures.

The differences in spatial extent were further reflected by the estimated areas of highly suitable habitats (cloglog ≥ 0.8) (Table 3). Fg-DON occupied the largest highly suitable area, reaching 2.669 million km^2^, followed by Fa-NIV (2.014 million km^2^), Fg-NIV (1.588 million km^2^), and Fa-DON (0.571 million km^2^). Thus, Fg-DON showed the largest potential environmental range, whereas Fa-DON represented the most geographically constrained group. The approximately five-fold difference in highly suitable area between Fg-DON and Fa-DON highlights substantial variation in potential geographic extent among FGSC groups.

Overall, current suitability predictions revealed pronounced within-complex differentiation among FGSC groups. The contrast between the widespread Fg-DON pattern, the geographically concentrated Fg-NIV pattern, and the Asia-centered but differently extended Fa-DON and Fa-NIV patterns demonstrates that FGSC suitability is structured by group-specific environmental associations. These results provide the spatial baseline for evaluating future climate-driven redistribution patterns among FGSC groups.

### 3.3. Future Suitability Responses and Spatial Redistribution Under Multiple Climate Scenarios

Future projections under four SSP scenarios and four future periods revealed divergent suitability trajectories among the four FGSC species-genotype groups (Table 3). Rather than exhibiting a consistent response to climate change, different groups showed distinct patterns of contraction, expansion, persistence, and spatial redistribution. These contrasting responses indicate that future changes in FGSC suitability are strongly group-specific.

**Figure 3:**
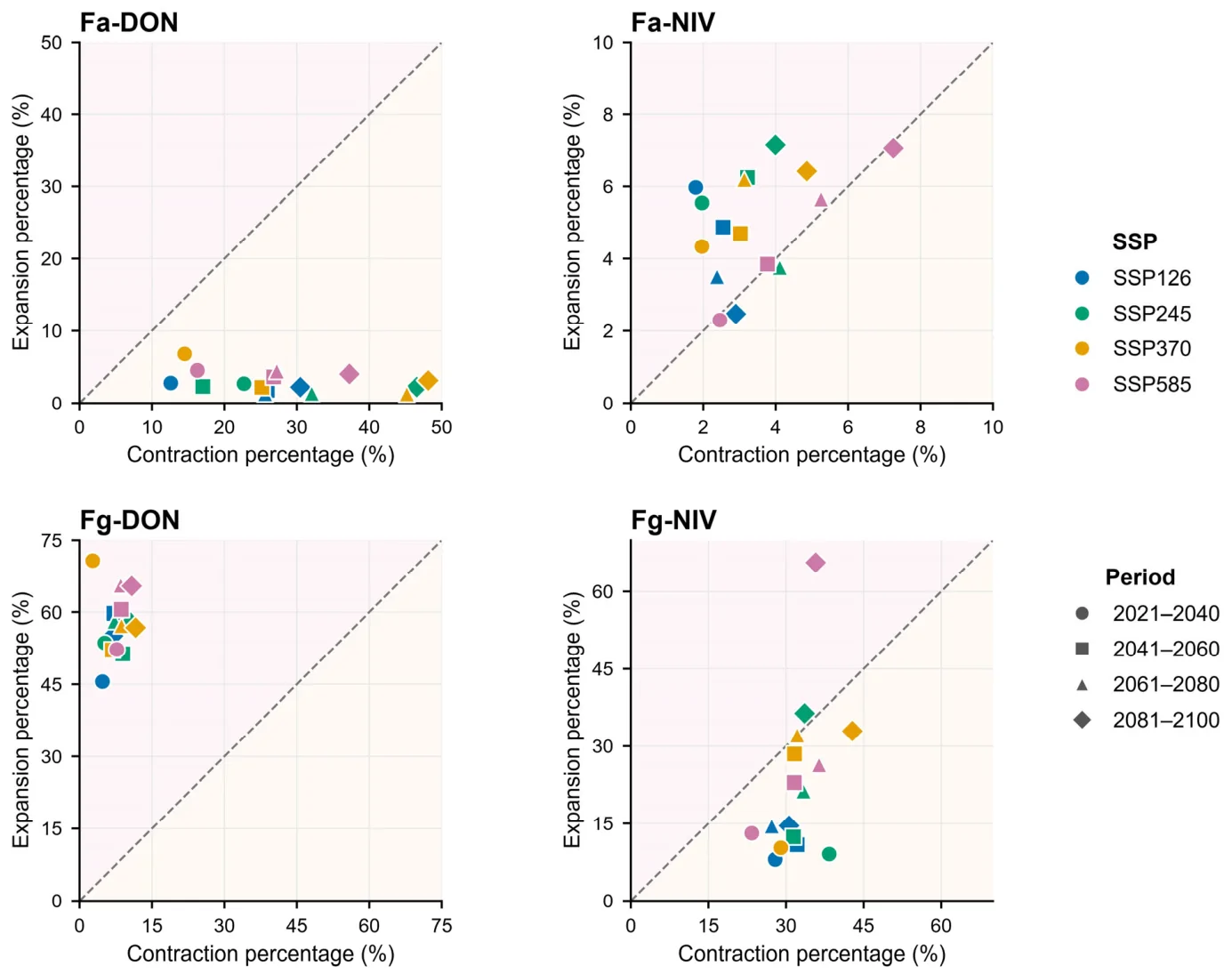
Projected balance between expansion and contraction of high-suitability areas under future climate scenarios. Shading above and below the dashed 1:1 line denotes net expansion and net contraction, respectively.

Fa-DON exhibited a consistent contraction pattern across future climate scenarios. Its highly suitable area decreased from 0.571 million km^2^ under current conditions to 0.314–0.527 million km^2^ across future projections, corresponding to an overall reduction of approximately 7.7–45.0%. The loss of suitable areas was mainly concentrated within its current distributional range, while newly gained suitable areas represented a relatively limited proportion of future changes (Figure 3). Continental-scale centroid analyses further showed that Fa-DON experienced relatively limited spatial displacement of suitable areas, suggesting that future changes were primarily characterized by regional contraction rather than large-scale redistribution (Table S4).

In contrast, Fg-DON displayed the strongest expansion response among the four groups. Its highly suitable area increased from 2.669 million km^2^ under current conditions to 3.757–4.483 million km^2^ under future scenarios, representing an increase of approximately 40.8–68.0%. The newly suitable areas were concentrated in specific latitudinal zones rather than being evenly distributed globally (Figure 3). Continental-scale analyses further indicated that Fg-DON expansion was accompanied by directional shifts of suitable-area centers in several continents, reflecting a combined process of spatial expansion and regional redistribution (Table S4).

Fa-NIV showed comparatively stable future suitability patterns. Across all SSP scenarios and future periods, its highly suitable area remained close to the current estimate of 2.014 million km^2^, ranging from 2.005 to 2.098 million km^2^. Continental centroid shifts likewise indicated relatively limited spatial reorganization for Fa-DON and Fa-NIV, whereas the two *F. graminearum* groups showed larger and more regionally variable shifts.

Fg-NIV exhibited pronounced scenario-dependent spatial reorganization. Its highly suitable area ranged from 1.123 to 2.063 million km^2^ across future projections, compared with 1.588 million km^2^ under current conditions. These values corresponded to changes ranging from a 29.3% contraction to a 29.9% expansion. Most projections indicated contraction or limited net change, whereas expansion occurred under SSP245 during 2081–2100 and was strongest under SSP585 during 2081–2100. These contrasting outcomes indicate that the future suitability of Fg-NIV is sensitive to both emission pathway and projection period.

Overall, future projections revealed four distinct response modes among FGSC species-genotype groups: contraction with limited redistribution in Fa-DON, broad expansion with directional redistribution in Fg-DON, spatial persistence in Fa-NIV, and strong scenario-dependent reorganization in Fg-NIV. These divergent trajectories demonstrate that climate change may reshape FGSC suitability through group-specific spatial processes rather than a uniform shift across the entire species complex.

### 3.4. Environmental Drivers Underlying Differential Suitability Patterns Among FGSC Species-Genotype Groups

**Figure 4:**
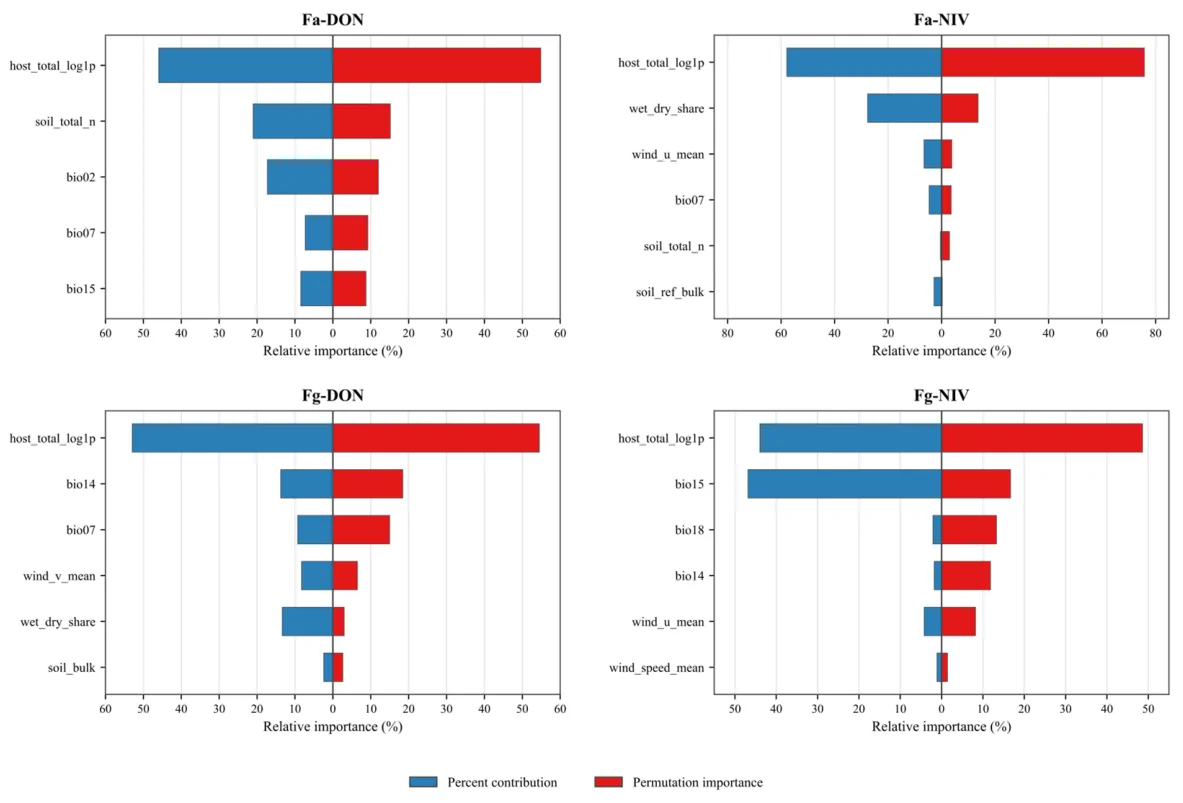
Relative importance of environmental variables for four FGSC groups.

Permutation importance analysis revealed that host-related variables represented the dominant environmental constraints shaping the potential suitability of all four FGSC species-genotype groups (Figure 4). Among all predictors, host_total_log1p consistently ranked as the most important variable across groups, with permutation importance values of 54.87%, 75.80%, 54.49%, and 48.60% for Fa-DON, Fa-NIV, Fg-DON, and Fg-NIV, respectively. These results indicate that the spatial availability of cereal host resources represents a shared ecological foundation influencing the suitability patterns of different FGSC groups.

Despite the consistent importance of host availability, secondary predictors differed substantially among groups. For Fa-DON, soil_total_n, bio02, bio07, and bio15 contributed additional information after host_total_log1p. Fa-NIV showed the strongest dependence on host-related predictors, with wet_dry_share providing complementary information beyond total host availability. Fg-DON retained a broader predictor structure, particularly bio14 and bio07. For Fg-NIV, host_total_log1p had the highest permutation importance (48.60%), followed by bio15 (16.67%), bio18 (13.27%), bio14 (11.82%), wind_u_mean (8.21%), and wind_speed_mean (1.42%).

**Figure 5:**
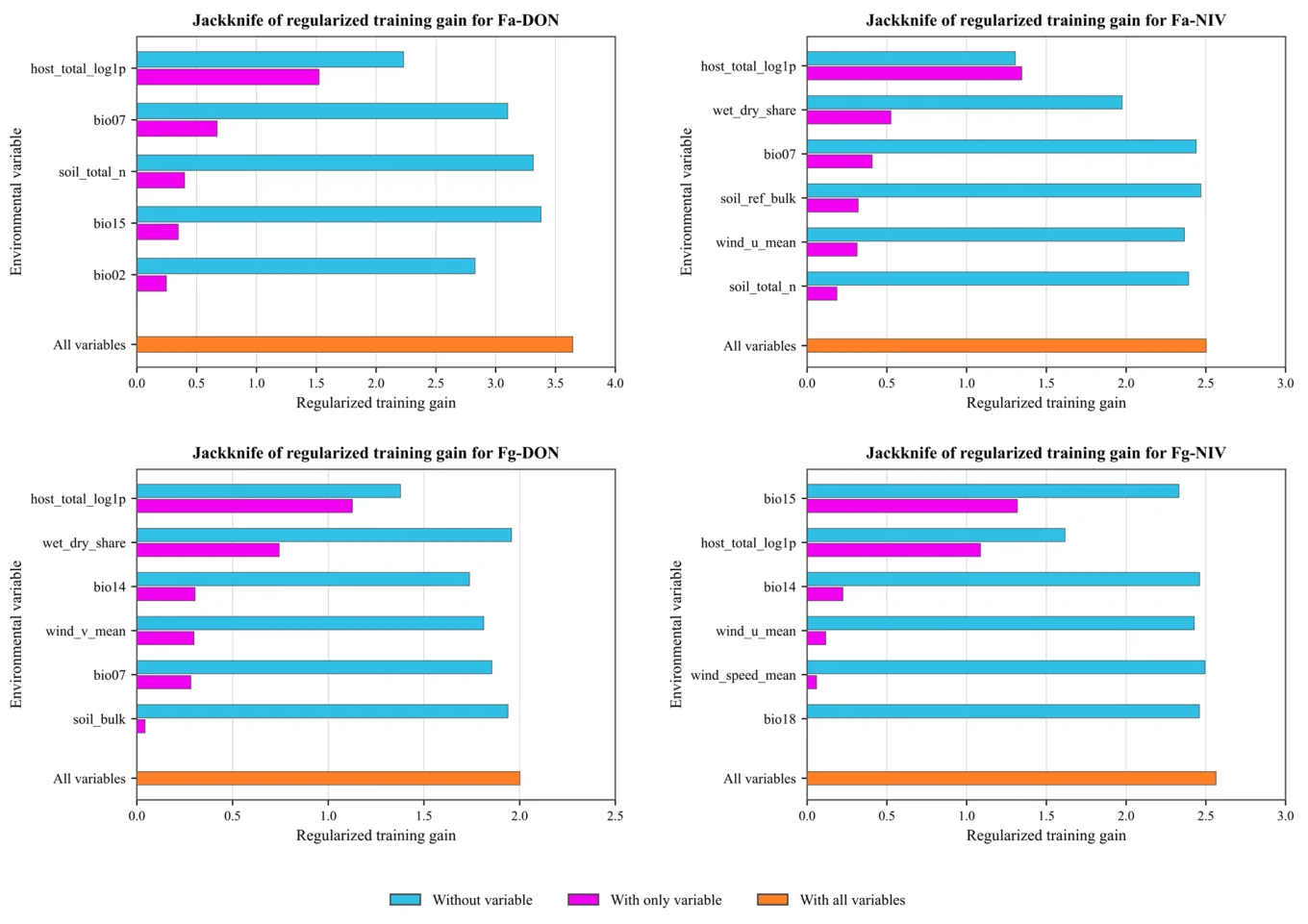
Jackknife test results of regularized training gain.

Jackknife analyses further distinguished independent from conditional predictor information (Figure 5). Across groups, host_total_log1p supplied substantial unique information, as shown by its gain when used alone and the reduction in gain when omitted. For Fa-NIV, wet_dry_share provided additional information beyond total host abundance. In the Fg-NIV model, bio18 yielded almost no training gain when used alone (0.0003), but omitting it reduced regularized training gain from 2.5635 to 2.4595. This pattern indicates that bio18 contributed mainly within the multivariable model rather than as a strong standalone predictor.

Response curves showed group-specific relationships between retained predictors and modeled suitability (Figures S3–S6). Host availability generally increased suitability, although response magnitude and saturation differed among groups. Climatic predictors showed distinct response shapes and ranges across groups. These response curves represent marginal model responses and should not be interpreted as single-factor causal effects. Together, permutation importance, jackknife analysis, and response curves showed that FGSC suitability reflected shared host-resource gradients and group-specific environmental constraints.

### 3.5. Robustness Checks Across Spatial Resolutions

To evaluate whether the major suitability patterns were sensitive to spatial resolution, additional analyses were conducted at 0.25° and 1° resolutions while retaining the predictor sets, feature classes, and regularization multipliers selected in the 0.5° baseline models. Model performance remained consistent across spatial resolutions, with spatial test AUC values ranging from 0.928 to 0.979 across the four FGSC species-genotype groups (Table S5). The minimum fold-specific test AUC values also remained above 0.90, indicating that changes in spatial aggregation did not substantially affect model discrimination.

The predicted suitability patterns showed strong spatial consistency among different resolutions. Compared with the 0.5° baseline predictions, alternative-resolution models exhibited high agreement in continuous suitability distributions, with Spearman correlation coefficients ranging from 0.823 to 0.986 and Schoener’s D values indicating substantial niche overlap among resolutions (Table S5). These results suggest that the major spatial structures of suitability patterns were preserved despite changes in spatial resolution.

However, estimates of highly suitable areas based on cloglog ≥ 0.8 showed greater variation among spatial resolutions. For example, the estimated highly suitable area of Fg-DON varied from 1.701 million km^2^ at 0.25° resolution to 2.669 million km^2^ at 0.5° and 4.217 million km^2^ at 1° resolution. Similar scale-dependent differences were observed for other FGSC groups (Table S5). This indicates that absolute area estimates derived from threshold-based classification are influenced by spatial aggregation, whereas the relative differences among FGSC groups and the overall spatial patterns remained stable.

Overall, resolution sensitivity analyses confirmed that the major ecological conclusions regarding group-specific suitability patterns and future suitability changes were robust across spatial scales. Nevertheless, absolute estimates of highly suitable area should be interpreted in the context of spatial resolution.

## 4. Discussion

This study revealed distinct high-suitability cores and future trajectories among the four FGSC species-genotype groups across four SSPs and four future periods. Specifically, (a) Fa-DON was concentrated along the middle and lower Yangtze River, while its future high-suitability area declined by 7.7–45.0%; (b) The highly suitable areas of Fa-NIV were located mainly across the Indo-Gangetic Plain and from southwestern China to the Yangtze River basin. Its projected area changed only slightly, from −0.4% to +4.2%; (c) Fg-DON had the broadest high-suitability range, spanning central and eastern North America, central-eastern Europe, the North China Plain, and southern South America. Its highly suitable area expanded by 40.8–68.0% across all scenario–period combinations; (d) Fg-NIV was concentrated in western-central Europe and eastern North America, with smaller suitable areas in southeastern Australia. Its projected change ranged from −29.3% to +29.9%. Median centroid displacement ranged from 99.0 to 229.8 km among groups, with relatively larger shifts for the two *F. graminearum* groups. The centroid of the highly suitable area of Fg-DON shifted mainly northwestward in Asia but southeastward in Europe and North America. The corresponding centroid for Fg-NIV shifted mainly northeastward in Europe and North America. These patterns suggest that future environmental change may produce regionally distinct adjustments in the modeled suitability of FGSC groups rather than a uniform complex-wide response.

Modeled suitability increased with host availability across all four groups, whereas responses to climatic, soil, and wind-related predictors differed in magnitude and shape. Host-related predictors contributed consistently to the modeled suitability patterns. host_total_log1p had the highest permutation importance in all four final models, ranging from 48.60% to 75.80%, indicating a strong model-based association between overall cereal-host availability and group-specific suitability. The wet_dry_share was also retained in the final models for Fa-NIV and Fg-DON, indicating that crop composition provided additional predictive information for these groups. Thus, host availability emerged as a shared correlate of modeled suitability, whereas crop composition and other environmental associations were more group-specific.

Previous projections have examined *Fusarium* distributions at species, genus, or regional disease-risk scales. A global climate-envelope analysis predicted suitable conditions for *F. graminearum* across most major rainfed wheat regions. In the same analysis, *F. asiaticum* was primarily associated with warm and humid regions of East Asia [47]. A global projection pooling *Fusarium* spp. predicted decreasing moderate and high suitability across parts of Australia and Africa [28]. It also projected suitability gains toward higher latitudes, including Russia and Canada. A regional FHB model for Korea projected increasing mean disease risk across suitable wheat-growing regions under future climates [33]. The four groups examined here followed more divergent trajectories. The predicted highly suitable area of Fg-DON expanded consistently, whereas that of Fa-DON contracted. Fa-NIV remained comparatively stable, while Fg-NIV varied among scenarios and periods. Consequently, trends derived from species- or genus-level projections may not fully resolve the responses of individual FGSC species-genotype groups.

Several factors may contribute to the differences between the present projections and previous studies. (a) Species- or genus-level models generally pool occurrence records across biological groups, producing an integrated estimate of their environmental niche. (b) Regional FHB models estimate disease risk for particular crops and phenological stages, which differs from environmental suitability for a species-genotype group. (c) The present study treated the four species–genotype groups as separate response units and independently selected predictors and tuning parameters for each group. The models incorporated climatic, host-related, soil, topographic, and wind predictors. This framework extends previous species- and genus-level projections by examining potential differences between DON and NIV groups within the same species. Such grouping is consistent with the established ecological and biogeographical heterogeneity of the FGSC [2,20]. Nevertheless, additional regional data will be needed to examine the predicted patterns further.

Group-specific suitability projections may support future surveillance in several ways: (a) Regions that consistently remain highly suitable may be considered for supplementary sampling and pathogen identification; (b) Regions showing substantial future changes may be prioritized for repeated surveys; (c) Regions with divergent DON and NIV projections may warrant closer examination of their species-genotype composition. These model outputs represent relative environmental suitability rather than observed occurrence frequency, regional dominance, disease severity, or mycotoxin contamination. Predicted high suitability therefore does not confirm the local presence of a particular group. Interpretation should incorporate local cropping systems, pathogen identification, field observations, and regional epidemiological evidence.

This study has several limitations. The occurrence records were compiled from published studies rather than a standardized global surveillance network. Available records were concentrated in East Asia, Europe, and North America, whereas coverage remained limited across parts of Africa, Oceania, and South America. Record density may therefore reflect actual distribution, research effort, sampling accessibility, and reporting bias [48]. Occurrence sample sizes also differed among groups, which may have produced unequal levels of predictive uncertainty. MaxEnt contrasts occurrence locations with background sites, so unrecorded locations do not represent confirmed absences. Future projections used 20-year mean environmental conditions rather than annual simulations. This temporal framework facilitates comparisons among SSPs and periods but reduces sensitivity to extreme events, interannual variability, and short-term phenological changes. The SPAM2020 host layers were held constant, so future changes in crop distributions, rotations, and management practices were not represented. Climate projections were derived from BCC-CSM2-MR alone, leaving uncertainty among global climate models unquantified. Future studies could integrate standardized surveillance, independent validation, multi-model climate ensembles, annual or seasonal climate data, and dynamic host and management scenarios.

## 5. Conclusions

This study used a host-informed MaxEnt framework to compare current suitability with future projections under four SSPs, while retaining SPAM2020 host conditions. The four FGSC species-genotype groups differed in their highly suitable areas, continental patterns, influential predictors, and projected responses. Fg-DON had the broadest current highly suitable area, whereas Fa-DON was the most spatially restricted. Across future projections, high-suitability area (Cloglog ≥ 0.8) expanded by 40.8–68.0% for Fg-DON and contracted by 7.7–45.0% for Fa-DON. Fa-NIV remained comparatively stable, while Fg-NIV showed scenario-dependent changes ranging from a 29.3% contraction to a 29.9% expansion. Centroid movements further indicated regional reorganization rather than a uniform poleward shift. Host availability was the most consistently influential predictor, while climatic, soil, topographic, and wind-related variables provided additional group-specific information. Spatial cross-validation and resolution sensitivity analyses supported the broad suitability patterns and major change directions, although absolute threshold-based area estimates remained scale-dependent. These projections provide a comparative reference for prioritizing FGSC surveillance and mycotoxin monitoring. However, they represent potential environmental suitability rather than realized occurrence, disease severity, or mycotoxin risk and should be applied together with regional field and epidemiological evidence.

## Figures and Tables

**Table 1:** Summary of datasets and corresponding scenarios sources used in this study.

Data Category	Purpose	Scenario	Source
WorldClim 2.1 (bio1-bio19, elevation)	Current climate and elevation predictors	Current baseline (as defined by WorldClim v2.1)	WorldClim 2.1 (https://worldclim.org/, accessed on 18 August 2026)
CMIP6 (BCC-CSM2-MR, SSP126; SSP245; SSP370; SSP585)	Future climate predictors	2021–2040; 2041–2060; 2061–2080; 2081–2100	CMIP6 (https://pcmdi.llnl.gov/CMIP6/, accessed on 18 August 2026)
HWSD 2.0 (20 soil variables)	Soil predictors	Static	FAO/IIASA (HWSD2.0) (https://www.fao.org/soils-portal/data-hub/soil-maps-and-databases/, accessed on 18 August 2026)
Slope	Topographic predictor (slope)	Static	EarthEnv (https://www.earthenv.org/, accessed on 18 August 2026)
Host-related crop predictors	Definition of host-related predictors	Static (SPAM2020 v2.2)	SPAM2020 v2.2 (https://www.mapspam.info/data/, accessed on 18 August 2026)
Wind-related layers	sfcWind, uas, and vas	Current and future (scenario-consistent)	CMIP6 (https://cds.climate.copernicus.eu/datasets/projections-cmip6?tab=download, accessed on 18 August 2026)

**Table 2:** Final MaxEnt model configurations and selected predictors for the four FGSC groups.

Species-Genotype Group	Number of Retained Predictors	Predictor Set	FC ^1^	RM ^2^	K ^3^	AICc ^4^	Retained Predictors
Fa-DON	5	P90	LQ	0.5	10	1728.627	host_total_log1p; soil_total_n; bio07; bio02; bio15
Fa-NIV	6	P90	LQHP	2.5	22	2266.767	host_total_log1p; soil_total_n; bio07; wind_u_mean; wet_dry_share; soil_ref_bulk
Fg-DON	6	P90	LQHPT	2.0	96	14,528.036	host_total_log1p; bio14; wind_v_mean; bio07; wet_dry_share; soil_bulk
Fg-NIV	6	P85	L	0.5	6	725.574	host_total_log1p; bio14; bio15; bio18; wind_u_mean; wind_speed_mean

^1^ FC, feature-class; ^2^ RM, regularization-multiplier; ^3^ K, number of effective (non-zero) model parameters; ^4^ AICc, Akaike information criterion.

**Table 3:** Current and future highly suitable areas (million km^2^).

FGSC Group	Scenario	Current	2021–2040	2041–2060	2061–2080	2081–2100
Fa-DON	SSP126	0.571	0.515	0.434	0.433	0.410
	SSP245	0.571	0.457	0.487	0.396	0.318
	SSP370	0.571	0.527	0.440	0.321	0.314
	SSP585	0.571	0.504	0.439	0.441	0.382
Fa-NIV	SSP126	2.014	2.098	2.061	2.036	2.005
	SSP245	2.014	2.086	2.075	2.007	2.078
	SSP370	2.014	2.062	2.048	2.075	2.045
	SSP585	2.014	2.011	2.015	2.022	2.010
Fg-DON	SSP126	2.669	3.757	4.073	3.981	3.954
	SSP245	2.669	3.960	3.800	4.026	3.928
	SSP370	2.669	4.483	3.879	3.964	3.874
	SSP585	2.669	3.857	4.055	4.191	4.126
Fg-NIV	SSP126	1.588	1.272	1.250	1.386	1.334
	SSP245	1.588	1.123	1.286	1.396	1.632
	SSP370	1.588	1.290	1.538	1.588	1.431
	SSP585	1.588	1.425	1.451	1.429	2.063

## Data Availability

The raw data and intermediate processing files are available from the corresponding author upon reasonable request and will be provided free of charge for academic and non-commercial research purposes.

## Supplementary Materials

The following supporting information can be downloaded at https://www.mdpi.com/article/10.3390/foods15173096/s1, Figure S1: Global distributions of retained 0.5° occurrence cells for the four FGSC species-trichothecene genotype groups; Figure S2: Receiver operating characteristic curves of the final MaxEnt models for the four FGSC species-trichothecene genotype groups; Figure S3: Bootstrap response curves of retained predictors for Fa-DON; Figure S4: Bootstrap response curves of retained predictors for Fa-NIV; Figure S5: Bootstrap response curves of retained predictors for Fg-DON; Figure S6: Bootstrap response curves of retained predictors for Fg-NIV; Table S1: Occurrence-record processing and retained binary occupied 0.5° cells for the four FGSC species-genotype groups; Table S2: Environmental predictors and supporting layers used in MaxEnt modeling of the four FGSC species-trichothecene genotype groups; Table S3: Four-fold spatial cross-validation performance of the final MaxEnt model configurations for the four FGSC species-trichothecene genotype groups; Table S4: Continental centroid shifts of highly suitable areas of the four FGSC species-trichothecene genotype groups under future climate scenarios; Table S5: Spatial-resolution sensitivity analysis of the final MaxEnt models at 0.25°, 0.5°, and 1°.

## Abbreviations

The following abbreviations are used in this manuscript:

FGSC	*Fusarium graminearum* species complex
DON	Deoxynivalenol
NIV	Nivalenol
Fa-DON	*Fusarium asiaticum*-DON
Fa-NIV	*Fusarium asiaticum*-NIV
Fg-DON	*Fusarium graminearum*-DON
Fg-NIV	*Fusarium graminearum*-NIV
MaxEnt	Maximum Entropy
AUC	Area under the curve
FC	Feature-class
RM	Regularization-multiplier
AICc	Akaike information criterion
