# Supplementary material for: Host-Informed MaxEnt Modeling Reveals Distinct Global Distribution and Redistribution Patterns of Four Major Fusarium graminearum Species-Genotype Groups Under Current and Future Climates
Source: Foods. 2026 Aug 31;15(17):3096. doi: 10.3390/foods15173096 (PMC13565135; doi:10.3390/foods15173096)
Supplement: Supplementary file 1 [file foods-15-03096-s001.zip › Figure S1.pdf]

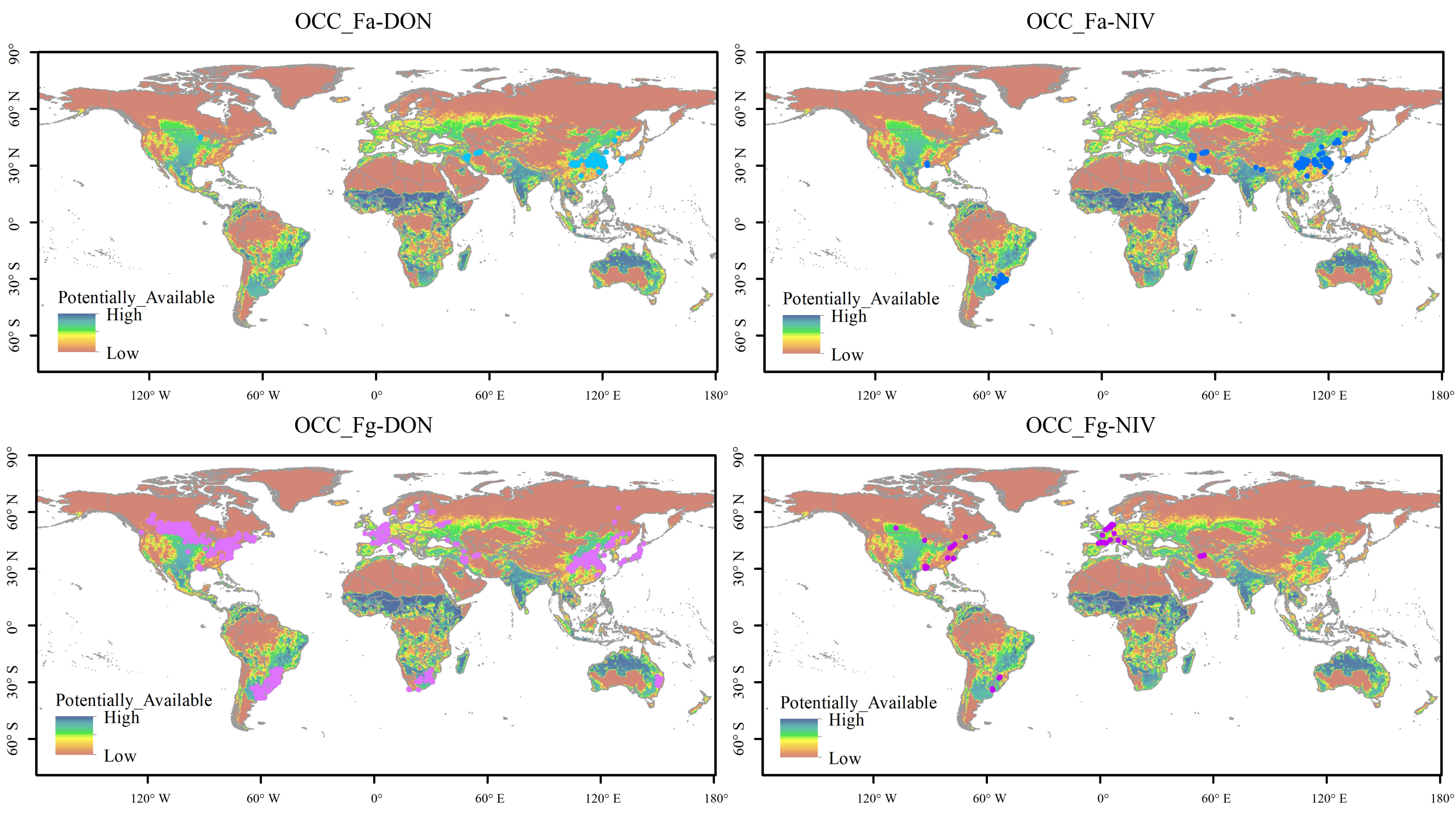
OCC_Fa-DON
OCC_Fa-NIV
OCC_Fg-DON
OCC_Fg-NIV
90°
60° N
30° N
0°
30° S
60° S
120° W
60° W
0°
60° E
120° E
180°
Potentially_Available
High
Low
