# Supplementary material for: Host-Informed MaxEnt Modeling Reveals Distinct Global Distribution and Redistribution Patterns of Four Major Fusarium graminearum Species-Genotype Groups Under Current and Future Climates
Source: Foods. 2026 Aug 31;15(17):3096. doi: 10.3390/foods15173096 (PMC13565135; doi:10.3390/foods15173096)
Supplement: Supplementary file 1 [file foods-15-03096-s001.zip › Figure S2.pdf]

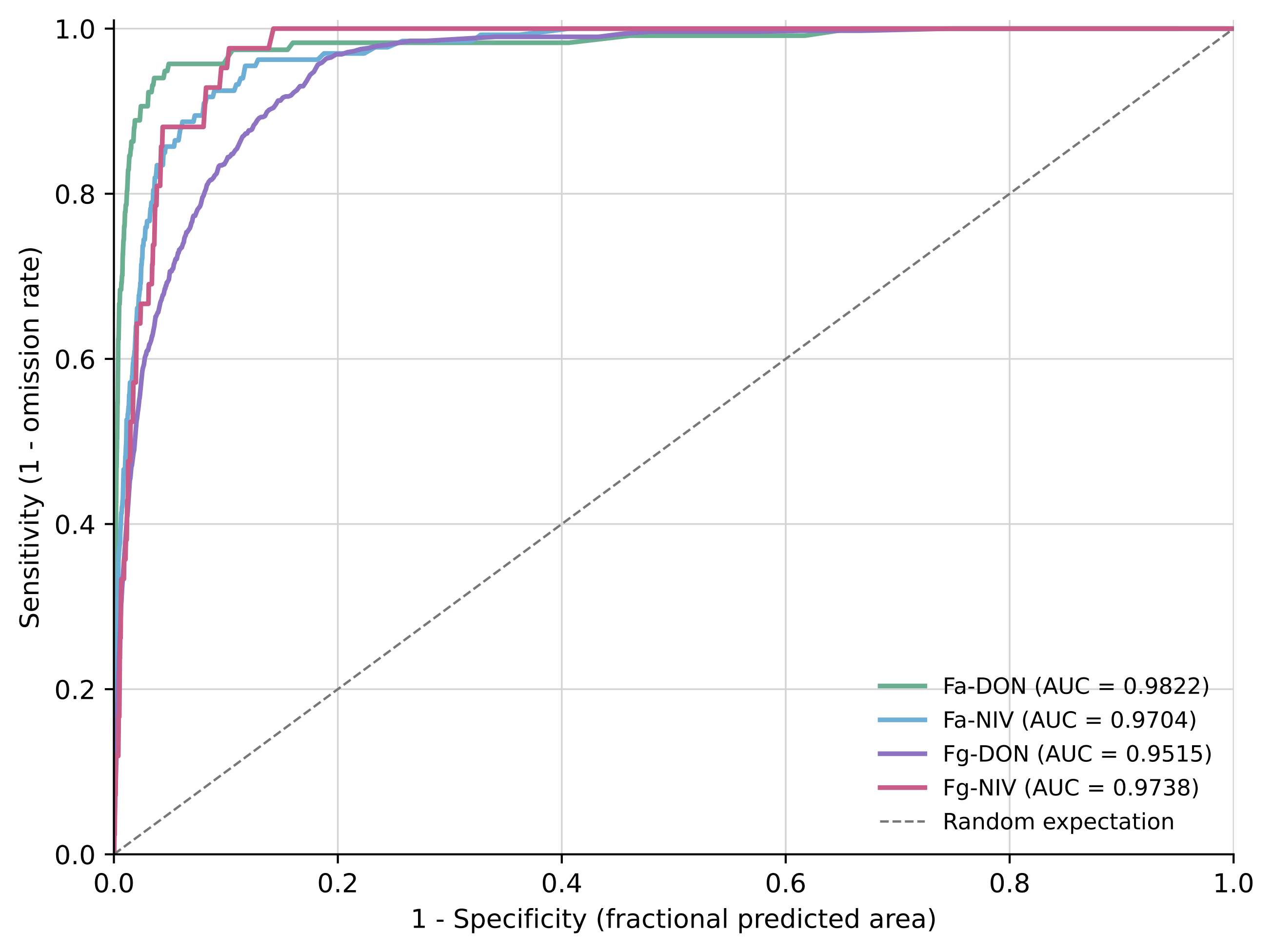

Sensitivity (1 - omission rate)
1 - Specificity (fractional predicted area)
0.0
0.2
0.4
0.6
0.8
1.0
Fa-DON (AUC = 0.9822)
Fa-NIV (AUC = 0.9704)
Fg-DON (AUC = 0.9515)
Fg-NIV (AUC = 0.9738)
Random expectation
