# Supplementary material for: Host-Informed MaxEnt Modeling Reveals Distinct Global Distribution and Redistribution Patterns of Four Major Fusarium graminearum Species-Genotype Groups Under Current and Future Climates
Source: Foods. 2026 Aug 31;15(17):3096. doi: 10.3390/foods15173096 (PMC13565135; doi:10.3390/foods15173096)
Supplement: Supplementary file 1 [file foods-15-03096-s001.zip › Figure S4.pdf]

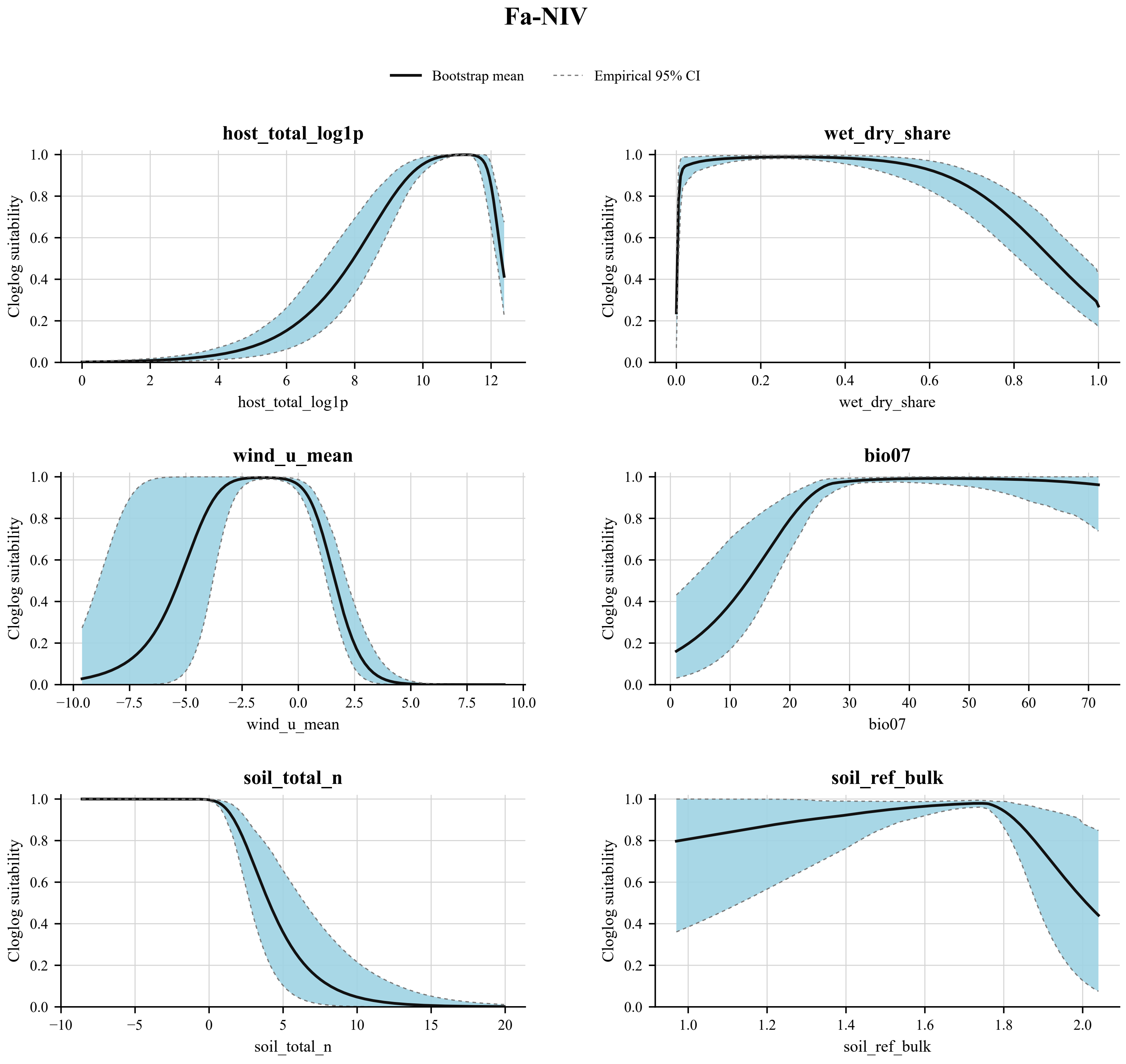

Fa-NIV
Bootstrap mean
Empirical 95% CI
host_total_log1p
wet_dry_share
wind_u_mean
bio07
soil_total_n
soil_ref_bulk
Cloglog suitability
