# Supplementary material for: Host-Informed MaxEnt Modeling Reveals Distinct Global Distribution and Redistribution Patterns of Four Major Fusarium graminearum Species-Genotype Groups Under Current and Future Climates
Source: Foods. 2026 Aug 31;15(17):3096. doi: 10.3390/foods15173096 (PMC13565135; doi:10.3390/foods15173096)
Supplement: Supplementary file 1 [file foods-15-03096-s001.zip › Figure S5.pdf]

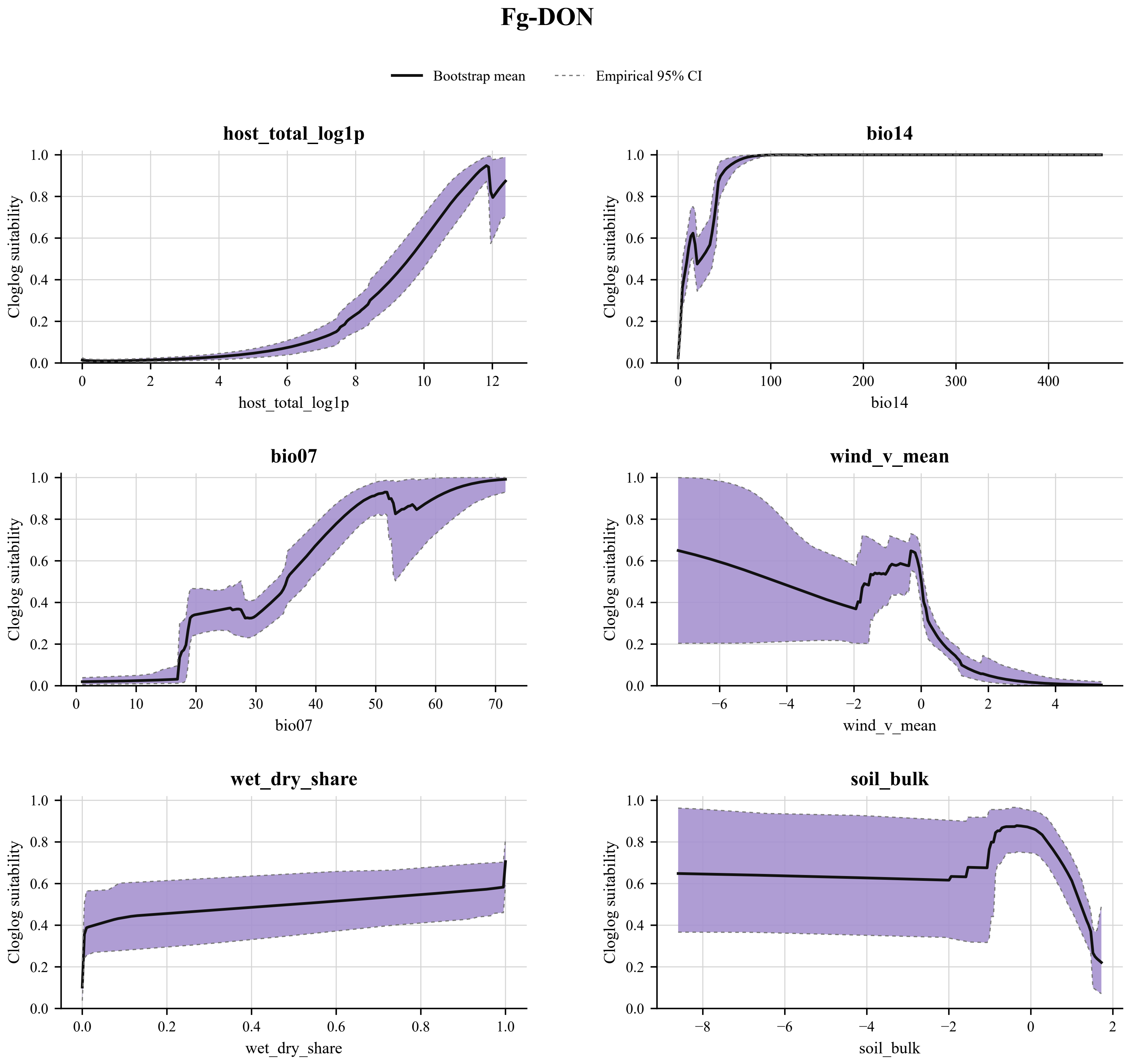
Fg-DON
Bootstrap mean
Empirical 95% CI
host_total_log1p
bio14
bio07
wind_v_mean
wet_dry_share
soil_bulk
Cloglog suitability
