# Supplementary material for: Host-Informed MaxEnt Modeling Reveals Distinct Global Distribution and Redistribution Patterns of Four Major Fusarium graminearum Species-Genotype Groups Under Current and Future Climates
Source: Foods. 2026 Aug 31;15(17):3096. doi: 10.3390/foods15173096 (PMC13565135; doi:10.3390/foods15173096)
Supplement: Supplementary file 1 [file foods-15-03096-s001.zip › Supplementary file legends.pdf]

Supplementary file legends

Supplementary Figures:

Figure S1. Global distributions of retained 0.5° occurrence cells for the four FGSC species-trichothecene genotype groups.

Figure S2. Receiver operating characteristic curves of the final MaxEnt models for the four FGSC species-trichothecene genotype groups.

Figure S3. Bootstrap response curves of retained predictors for Fa-DON.

Figure S4. Bootstrap response curves of retained predictors for Fa-NIV.

Figure S5. Bootstrap response curves of retained predictors for Fg-DON.

Figure S6. Bootstrap response curves of retained predictors for Fg-NIV.

Supplementary Tables:

Table S1. Occurrence-record processing and retained binary occupied 0.5° cells for the four FGSC species-trichothecene genotype groups.

Table S2. Environmental predictors and supporting layers used in MaxEnt modeling of the four FGSC species-trichothecene genotype groups.

Table S3. Four-fold spatial cross-validation performance of the final MaxEnt model configurations for the four FGSC species–trichothecene genotype groups.

Table S4. Continental centroid shifts of highly suitable areas of the four FGSC species-trichothecene genotype groups under future climate scenarios.

Table S5. Spatial-resolution sensitivity analysis of the final MaxEnt models at 0.25°, 0.5°, and 1°.
